# Nutraceuticals for Diabetic Retinopathy: Recent Advances and Novel Delivery Systems

**DOI:** 10.3390/nu16111715

**Published:** 2024-05-30

**Authors:** Xiaoyuan Ye, Nicholas Siu Kay Fung, Wai Ching Lam, Amy Cheuk Yin Lo

**Affiliations:** 1 Department of Ophthalmology, The University of Hong Kong, Hong Kong 999077, China; yexy9@connect.hku.hk (X.Y.); nfung@hku.hk (N.S.K.F.); dr.waiching.lam@gmail.com (W.C.L.); 2 Department of Ophthalmology, University of British Columbia, 2550 Willow Street, Room 301, Vancouver, BC V5Z 3N9, Canada

**Keywords:** blindness, diabetes, dietary supplement, inflammation, nanotechnology, natural product, neovascularization, oxidative stress, retina, vessel

## Abstract

Diabetic retinopathy (DR) is a major vision-threatening disease among the working-age population worldwide. Present therapeutic strategies such as intravitreal injection of anti-VEGF and laser photocoagulation mainly target proliferative DR. However, there is a need for early effective management in patients with early stage of DR before its progression into the more severe sight-threatening proliferative stage. Nutraceuticals, natural functional foods with few side effects, have been proposed to be beneficial in patients with DR. Over the decades, many studies, either in vitro or in vivo, have demonstrated the advantages of a number of nutraceuticals in DR with their antioxidative, anti-inflammatory, neuroprotective, or vasoprotective effects. However, only a few clinical trials have been conducted, and their outcomes varied. The low bioavailability and instability of many nutraceuticals have indeed hindered their utilization in clinical use. In this context, nanoparticle carriers have been developed to deliver nutraceuticals and to improve their bioavailability. Despite its preclinical nature, research of interventive nutraceuticals for DR may yield promising information in their clinical applications.

## 1. Introduction

Diabetic retinopathy (DR) is one of the microvascular complications of diabetes mellitus. It is a major cause of blindness worldwide especially among working-age adults, making it an extensive burden for both families and countries [1,2,3]. It was estimated that DR affects about 35% of diabetic patients globally [4].

DR starts as non-proliferative DR (NPDR) and progresses to proliferative DR (PDR). In NPDR, microaneurysms, small hemorrhages, hard exudates, cotton wool spots, venous beadings, and intraretinal microvascular abnormalities can be observed during ophthalmic examination. Later, new vessels growing toward the vitreous cavity from the retina occur in PDR [5]. Vitreous hemorrhage and tractional retinal detachment often happen in the later stages of PDR. Diabetic macular edema (DME) is another severe manifestation and can arise at any stage of DR. Based on the impact on vision, mild and moderate NPDR are categorized as non-vision-threatening DR, while vision-threatening DR (VTDR) includes severe NPDR, PDR, and DME [2].

## 2. Pathophysiology of DR

Hyperglycemia in diabetes triggers oxidative stress and inflammation through multiple pathways, which, in turn, lead to the development of DR. Through the tricarboxylic acid (TCA) cycle, a high level of glucose causes overproduction of electron donors (nicotinamide adenine dinucleotide (NADH) and flavin adenine dinucleotide (FADH2)), resulting in increased proton gradient across the inner mitochondrial membrane [6]. The overproduction of reactive oxygen species (ROS) exposes the retina to oxidative stress. Hyperglycemia-induced oxidative stress is heavily involved in the pathology of DR. The increase in the upstream glycolytic metabolites also induces (i) the accumulation of the advanced glycation end products (AGEs) and the activation of the receptor for AGEs (RAGEs); (ii) the activation of protein kinase C (PKC); (iii) the increased hexosamine pathway flux; and (iv) the active polyol pathway, a two-step metabolic pathway where glucose is reduced to sorbitol and then to fructose [7,8,9]. The disturbance of these four metabolic pathways, in turn, aggravates oxidative stress [10].

Oxidative stress not only augments the aforementioned metabolic pathways but also causes mitochondrial damage, cell death, inflammation, and lipid peroxidation in the retina. Notably, lipids make up one-third of the retina’s dry weight, of which approximately 40% are saturated fatty acids (SFA), 21% are monounsaturated fatty acids (MUFA), and 35% are polyunsaturated fatty acids (PUFA) [11,12]. Due to the sensitivity of PUFAs to oxidation, the retina exhibits high vulnerability to oxidative stress, making lipid peroxidation an important contributor to the progression of DR [10]. In the presence of oxygen-derived free radicals, lipid is oxidized and the products, such as hydroxyhexenal and hydroxynonenal, can interact with cellular DNA and proteins in the retina, leading to the damage of retinal cells like photoreceptors and retinal pigment epithelial (RPE) cells [13,14].

Inflammation is also crucial in the development of DR. Persistent inflammation triggers the activation of various immune cells, including microglia and macrophages [15]. Upregulation of pro-inflammatory chemokines such as interleukin (IL)-8, IL-1β, and tumor necrosis factor (TNF)-α have been detected in the aqueous and vitreous fluid as well as the serum of patients with PDR [16,17,18,19]. The pivotal mediators of inflammatory responses, nuclear factor-kappa B (NF-κB) and NOD-like receptor family pyrin domain containing 3 (NLRP3) inflammasomes are also highly related to the progression of DR [20,21,22,23]. Inflammation partly contributes to pericyte depletion and leukostasis, the adhesion of leukocytes to the retinal vasculature [24]. Leukostasis can, in turn, lead to injury and death of endothelial cells, nonperfusion of capillaries, ischemia, and disruption of the blood–retinal barrier (BRB), resulting in retinal vascular leakage [25]. Meanwhile, the subsequent retinal nonperfusion and ischemia not only further exacerbate oxidative stress, and promote the release of pro-inflammatory factors, but also induce proangiogenic factors like vascular endothelial growth factor (VEGF) and angiopoietin-2 (Ang-2) [26,27,28,29]. The process of VEGF-mediated angiogenesis ultimately leads to the progression of NPDR to PDR [30].

Emerging evidence indicates that neurodegeneration in the retina is another major characteristic of DR. Functional studies using multifocal electroretinography reported reduced amplitudes and increased peak implicit times in a proportion of patients with DR, suggesting a role of neuro-retinal dysfunction in the pathogenesis of DR [31,32,33]. Besides functional changes, the peripapillary retinal nerve fiber layer has been detected to be significantly thinner in diabetic patients compared with controls [34]. Research using animal models that mimic DR has consistently demonstrated a significant involvement of functional impairment and cell death of retinal ganglion cells (RGCs), amacrine cells, and photoreceptors [35,36,37].

## 3. Treatments for DR

Currently, the primary focus of intervention is on the vision-threatening stage of DR: PDR and DME. Panretinal photocoagulation (PRP) is the mainstay treatment and is considered the standard care for PDR [5]. Intravitreal injection of anti-VEGF (e.g., Ranibizumab and Aflibercept) is a developing therapy and could be considered for additional treatment in patients with high-risk PDR [38]. For the central-involved DME, intravitreal anti-VEGF has become the first-line therapy as recommended in 2019′s Preferred Practice Pattern by the American Academy of Ophthalmology (AAO) and 2022′s evidence-based guidelines in China [38,39]. Steroids such as intravitreal triamcinolone acetonide (IVTA) are suitable and can be tailored for patients with PDR or DME [5]. Recently, intravitreal Faricimab, the bispecific antibody that binds to and inhibits both vascular VEGF-A and Ang-2, has been approved to treat DME in numerous countries and regions including America, Europe, China, and Japan [40,41,42]. Despite the advances in treatments of PDR, management for NPDR is still limited; it primarily focuses on glycemic and blood pressure control as well as lipid-lowering strategies [5]. In this context, nutraceuticals have been proposed to be a promising adjunctive treatment that offers potential curative benefits for patients with NPDR, and even PDR.

## 4. Nutraceuticals

Nutraceuticals are natural functional foods that provide medical or health benefits. Some nutraceuticals, such as carotenoids, are strong antioxidants with the potential to scavenge the ROS. Some exert anti-inflammatory effects and may regulate the expression of NF-κB [43]. Some nutraceuticals have been shown to be advantageous to both neuronal and microvascular functions [44]. As natural foods, nutraceuticals are easily accessible and affordable. Moreover, proper dietary supplementation of nutraceuticals is considered safe and less likely to cause side effects that are commonly associated with many anti-diabetic medications, such as hypoglycemia, liver and kidney damage, and gastric discomfort.

**Figure 1:**
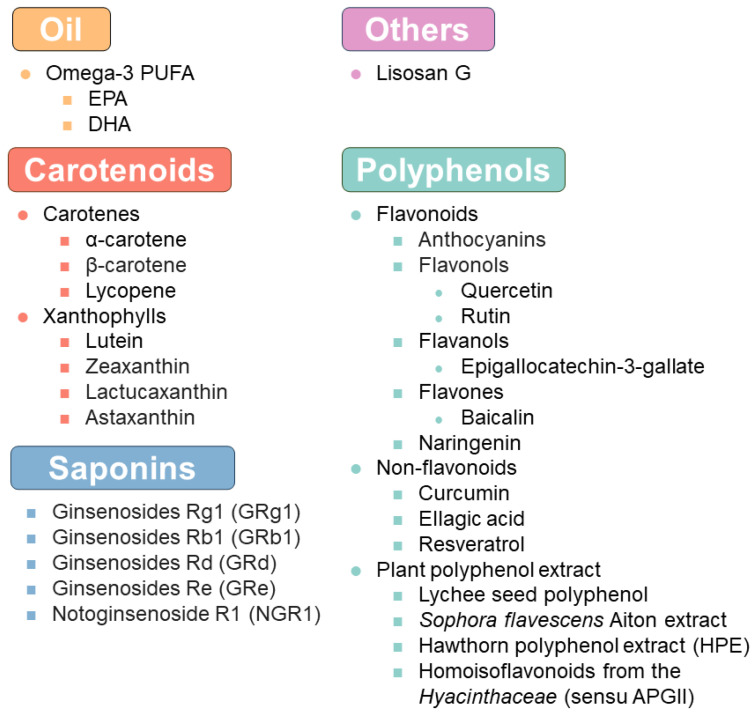
A summary of the nutraceuticals mentioned in the present review.

The role of nutraceuticals in DR was reviewed a few years ago [45,46]. Here, we focused on providing an update on the evidence of nutraceutical intervention in DR (Figure 1 and Table 1 and Table 2). Furthermore, we discussed the advances in delivery systems for these nutraceuticals that may help to resolve the bioavailability issues.

The search strategy and selection criteria were as follows: we searched Google Scholar and PubMed for original research articles as well as meta-analysis/systematic reviews. The research terms were “diabetic retinopathy”, “nutraceuticals”, or “delivery system” in combination with the term of a specific nutraceutical (e.g., lutein) or delivery system (e.g., liposomes). We chose publications within the last 5 years (2019 to March 2024) to illustrate recent advances. However, common references and important older publications were also included to provide a more comprehensive overview. Review articles, book chapters, and clinical guidelines were cited to provide readers with more detailed information.

### 4.1. Oil

Diabetic dyslipidemia is very common among patients with type 2 diabetes mellitus (T2DM), with a prevalence of 72–85% [47]. Docosahexaenoic acid (DHA), oleic acid (OA), and arachidonic acid (AA) are the “primary PUFAs” in the photoreceptor outer segments of the human retina [48]. These PUFAs are not only crucial for the structure, homeostasis, and function of the retina but are also effective antioxidants and anti-inflammatory substances [49,50,51]. A combined cohort study analyzed the association between omega-3 PUFAs and DR using data from the Multi-Ethnic Study of Atherosclerosis (MESA) and Genetics of Latino Diabetic Retinopathy (GOLDR). It concluded that DHA was negatively linked to the occurrence and severity of DR [52].

Another potential indicator of the severity of DR is the ratio of omega-6 (ω-6)/omega-3 (ω-3) fatty acids. Some of these fatty acids are metabolized by the same enzymes and are, therefore, competitors in the metabolic pathways [53]. It has been suggested that the ω-6/ω-3 ratio was more important than individual indicators [54]. A low ω-6/ω-3 ratio is generally considered to have a protective effect against certain chronic diseases such as coronary heart disease, autoimmune diseases, and diabetes by reducing the production of pro-inflammatory mediators [55,56,57]. However, in 2021, Zhao et al. reported that the ω-6/ω-3 ratio was positively related to the incidence of DM, but higher serum ω-6/ω-3 ratio was inversely associated with DR in a case–control study [58]. The authors explained that DM is a complex condition of metabolism and changes in ω-6/ω-3 ratio might not be a straightforward process that only increases with disease progression and can be affected by the composition of individual PUFAs [58]. In fact, it has been noted that both ω-3 and the ω-6 lipid products of cytochrome P450 oxidase 2C were shown to aggravate retinal and choroidal neovascularization in mice [59,60]. Therefore, the correlation between ω-6/ω-3 ratio and DR may be intricate.

Nevertheless, the idea of applying lipids as a source of diet supplements to aid DR has been proposed for years. Fish oil, soybean oil, and olive oil have been proposed as nutritional recommendations for the public due to their high proportion of healthy components such as PUFAs. Indeed, many preclinical evaluation experiments conducted in animal models have shown that supplementation of omega-3 PUFAs or DHA alone exerted protective effects in DR. Dietary PUFAs resulted in a beneficial effect on pathological retinal neovascularization in the mouse oxygen-induced retinopathy (OIR), an animal model of DR [61]. Here, DHA, and its enzymatic oxidation products (4-hydroxy-docosahexaenoic acid) as well as some bioactive omega-3 PUFA-derived mediators (neuroprotectinD1, resolvinD1 and resolvinE1) were found to have anti-angiogenic effects [61,62,63]. Several observational clinical studies also suggested a potential beneficial role of omega-3 PUFA-enriched diets such as fish-originated long-chain omega-3 PUFAs in lowering the risk of VTDR [64,65,66]. In a randomized controlled trial (RCT) in patients with DME, intravitreal ranibizumab combined with DHA supplementation was shown to reduce central subfield macular thickness but did not improve the visual acuity as compared with ranibizumab alone after a 2-year follow-up [67]. Despite these findings, a prospective clinical study (PAOXRED study) showed that intake of 1050 mg/day DHA for 2 years did not appear to mitigate NPDR progression [68]. Likewise, a prospective clinical study (ASCEND-Eye) found that daily oral supplementation of 1 g omega-3 fatty acids [containing 460 mg Eicosapentaenoic acid (EPA) and 380 mg DHA] for 6.5 years did not yield better outcomes versus control in DR progression [69]. Overall, the effect of PUFA supplements on DR remains ambiguous. PUFA-rich diets may be beneficial but may have a less significant effect, especially in vision improvement. PUFA may provide more of a supportive beneficial effect than a therapeutic one.

### 4.2. Carotenoids

Carotenoids are organic pigments mainly produced by plants or algae. They include two groups: carotenes (hydrocarbon carotenoids) and xanthophylls (oxygen-containing carotenoids). Alpha-carotene and beta-carotene are provitamin A carotenoids that can be converted to vitamin A via the beta-carotene monooxygenase type 1 (BCMO1) enzyme in the intestine [70]. Vitamin A is a family of fat-soluble retinoic acids, chiefly retinol and retinyl esters. One active derivative of vitamin A, 11-*cis*-retinal, is a critical component of rhodopsin that contributes to light perception [71]. Lutein, zeaxanthin, and meso-zeaxanthin are xanthophylls presented as macular pigments in the human eyes and contribute to absorbing damaging visible light and quenching ROS [72]. Lutein and zeaxanthin cannot be synthesized de novo and must be obtained from the diet. Meso-zeaxanthin is a metabolite of lutein but can also be absorbed from the diet. Carotenoids have been utilized as dietary supplementations for decades due to their importance in the eyes [73].

Some studies have revealed a relationship between decreased levels of carotenoids and the risk of DR. It was reported that the mean plasma levels of carotenoids were significantly lower in the DR group compared with the control group and DM patients without DR [74,75,76]. In in vitro or in vivo models, oral supplementation of lutein was demonstrated to have antioxidative, anti-inflammatory, and anti-angiogenic effects; it helps to rescue the retinal morphological changes caused by hyperglycemia [77,78,79]. Lycopene is another carotene that can inhibit oxidative damage to DNA, lipids, and proteins [80]. Oral intake of lycopene has been shown to be effective in preventing elevated inflammation and oxidative stress in the alloxan-induced diabetic eye tissues, the optic nerve in particular [81]. Astaxanthin, a xanthophyll produced by algae and found in many marine organisms, has sparked widespread interest among scientists in managing DR. Astaxanthin, either administered orally, intraperitoneally, or by intravitreal injection, is reported to suppress oxidative stress, downregulate inflammation, alleviate apoptosis, and help to improve visual acuity and control blood glucose via the phosphoinositide 3-kinase (PI3K)/Akt/nuclear factor erythroid 2-related factor 2 (Nrf2) pathway and hypoxia-inducible factor alpha (HIF-1α) pathway [82,83,84,85,86,87,88,89]. Another xanthophyll, lactucaxanthin is a carotenoid from lettuce. Lately, there is evidence to show that lactucaxanthin administered through oral consumption could modulate ROS and downregulate the expression of angiogenesis markers such as VEGF and HIF-1α. It exhibited the greater potential of being a bioactive anti-angiogenic nutrient supplement for DR when compared to lutein [90,91].

Carotenoid-rich natural plants have attracted widespread attention, with the belief that they have fewer side effects. Lutein-rich purple sweet potato leaf extract has been shown to suppress retinal inflammation and increase retina thickness while palm oil mill effluent-derived beta-carotene could alleviate oxidative stress as well as regulate BRB function in diabetic rats [92,93]. However, high doses of carotenoid supplementation may not benefit retinal function. A high-dose carotenoid-enriched carrot powder diet was reported to exacerbate retina function in streptozotocin (STZ)-induced diabetic rats [94]. It was found that the hepatic metabolism of carotenoids into retinoids in diabetic rats was lower compared with the non-diabetic rats, which partially contributed to this phenomenon [94]. Indeed, different delivery methods, dosage levels, administration durations, and metabolic statuses may lead to different results. Likewise, the outcomes after carotenoid supplementation are inconsistent in clinical trials [95,96,97]. However, two recent systematic reviews concluded that carotenoids may delay the onset and impede the development of DR as an adjunctive nutritional strategy, but they also stressed the need for longer, high-quality, placebo-controlled, and large-scale prospective clinical trials [98,99]. In addition, the lipophilic nature of carotenoids, as well as their absorption properties in the gastrointestinal tract, causes relatively low bioavailability with conventional pharmaceutical packaging and delivery methods. Therefore, further studies on the packaging of carotenoids and improvement of administration approaches are in demand.

### 4.3. Polyphenol

Polyphenols are found in many plant foods like fruits, vegetables, tea, coffee, and wine. Polyphenols can be broadly classified into four subgroups, including phenolic acids, flavonoids, stilbenes, and lignans [100]. Characterized by the presence of multiple phenol units or phenolic rings, polyphenols exhibit strong antioxidant capabilities. Numerous studies have demonstrated that dietary polyphenols hold many potential health attributes like anti-inflammatory, anti-carcinogenic, and anti-diabetic effects, preventing the development of chronic diseases like cardiovascular diseases and neurodegenerative disorders [101,102]. Here, we briefly introduced the roles of several polyphenols in DR.

#### 4.3.1. Flavonoids

Flavonoids are the most abundant and versatile class of polyphenols, featuring a common C6-C3-C6 backbone structure. Flavonoids can be further divided into several subcategories, including anthocyanidins, flavonols, flavanols (catechins and proanthocyanidins), flavones, isoflavones, flavanones, and chalcones [103].

- Anthocyanins

Anthocyanins are natural water-soluble pigments found inside the vacuoles in the cytosol and they contribute to the reddish-blue color of many fruits and vegetables. Black raspberry-derived anthocyanins (BRAs) have been extensively studied and demonstrated to regulate Nrf2 gene expression, attenuate ROS levels, reduce caspase-1-induced inflammation, and lower VEGFA levels, resulting in the melioration of blood glucose, lipid dysmetabolism, and retinopathy in diabetic rats [104,105]. Cyanidin-3-glucoside (C3G), also called chrysanthemin, is one of the most widely distributed anthocyanins found in nature. Similar to BRAs, C3G showed protective effects in in vivo and in vitro models of DR but gained more attention for its relative stability and easier absorbance [106,107,108]. Molecular docking analysis revealed that C3G could combine well with 78-kDa glucose-regulated protein (GRP78), a major endoplasmic reticulum (ER) chaperone protein, hence regulating ER stress [105,109]. In a recent open-label clinical trial, oral supplementation of 320 mg/day of anthocyanins for 4 weeks significantly reduced the pro-inflammatory biomarkers (IL-6, IL-18, and TNF-α) in T2DM patients [110]. Such evidence suggests that anthocyanins are a valuable dietary supplement for individuals with diabetes or DR.

- Flavonols

Quercetin is a flavonol and a plant pigment. Unlike anthocyanins, quercetin is insoluble in cold water, poorly soluble in hot water, but quite soluble in alcohol and lipids [111]. It is a strong antioxidant with five hydroxyl groups (-OH). Quercetin also exerts potent anti-inflammatory effects in DR through downregulating the level of NLRP3 inflammasomes and NF-κB signaling [112,113]. It was shown to inhibit autophagy and apoptosis and alleviate ER stress, therefore limiting pathological angiogenesis [112,113,114,115]. By upregulating nerve and synapse-associated proteins like postsynaptic density (PSD) 93, PSD95, nerve growth factor (NGF), and brain-derived neurotrophic factor (BDNF), quercetin is considered to be neurotrophic and neuroprotective in DR [113,114]. Interestingly, it was reported that oral consumption of quercetin not only regulated the Nrf2 pathway but also helped to reconstruct microbial community structure and inhibited intestinal dysbiosis, protecting retinal function through the gut–retina axis in DR [116]. Together, these studies indicate that quercetin is a functional nutraceutical that is helpful for DR.

Rutin, also known as quercetin-3-O-rutinoside, has identical antioxidant properties and demonstrates therapeutic potential for DR. Oral administration of rutin in STZ-induced DR rats resulted in a lower level of VEGF and a significantly lower tortuosity index [117].

- Flavanols

Flavanols, also called flavan-3-ols, are commonly found in foods like tea, cocoa, grapes, apples, and berries. Flavanols contain several compounds like catechin, epicatechin, epigallocatechin, epicatechin gallate, and epigallocatechin gallate. Epigallocatechin-3-gallate (EGCG) is the most abundant and bioactive catechin in green tea. A cross-sectional clinical study suggested that long-term tea consumption was significantly associated with a lower risk of DR [118]. Concordantly, EGCG was shown to stimulate autophagy and reduce apoptosis in the high glucose (HG)-treated rat primary retinal Müller cells [119]. Peracetate-protected (e)-EGCG (pro-EGCG) is a prodrug of EGCG with enhanced stability and bioavailability. Pro-EGCG was reported to be anti-inflammatory and could mitigate cell proliferation and pro-angiogenic factor production through the ROS/Thioredoxin-interacting protein (TXNIP)/NLRP3 inflammasome axis [120]. Another catechin, catechin 7-O-β-D-apiofuranoside (C7A), the main bioactive component of *Ulmus davidiana* extract, was demonstrated to prevent pericyte apoptosis and reduce endothelial permeability, suggesting its potential as a protective agent for reducing vascular leakage in DR [121].

- Naringenin

Naringenin is a natural flavonoid found primarily in citrus fruits such as tomatoes and oranges. Naringenin is a strong antioxidant and was shown to upregulate the level of endothelial nitric oxide synthase (eNOS), downregulate the level of ROS, and inhibit apoptosis in HG-insulted human retinal endothelial cells (HREC) [122]. In a study using an HG-induced DR zebrafish model, naringenin could regress glucose levels and reduce oxidative stress, normalize VEGF overexpression, and finally inhibit macular degeneration of early DR in zebrafish [123].

#### 4.3.2. Non-Flavonoids

- Curcumin

Curcumin is the principal active polyphenolic compound of the turmeric plant (*Curcuma longa*). The effects of curcumin on DR have been widely explored. Curcumin could promote the Nrf2/Heme Oxygenase-1 (HO-1) signaling process through extracellular signal-regulated kinase 1/2 (ERK1/2) activation, hence inhibiting HG-induced injury in ARPE-19 retinal pigment epithelial cells and improving retina morphology in STZ-treated rats [124,125]. In addition, it was reported that curcumin could inhibit the PI3K/Akt/mammalian target of the rapamycin (mTOR) signaling pathway, suppressing inflammation in HG-treated ARPE-19 retinal pigment epithelial cells [126]. A prospective clinical trial evaluated the effects of a fixed formula with curcumin (200 mg), artemisia (80 mg), bromelain (80 mg), and black pepper (2 mg) on DR patients [127]. The researchers found that the formula could restore central retinal thickness, visual acuity, and vessel density in T2DM patients with or without DME [127]. These findings provided the rationale for curcumin to serve as a functional supplementation for the management of DR in the healthcare setting.

- Resveratrol

Chemically, resveratrol is a stilbene derivative containing two isomeric forms: *cis*-resveratrol and *trans*-resveratrol, with the latter being the more biologically active and widely studied form. Resveratrol serves as an effective scavenger of free radicals and a modulator of various cellular signaling pathways [128]. In the context of DR, resveratrol is involved in multiple pathways that regulate oxidative stress, apoptosis, and inflammation, such as mitogen-activated protein kinases (MAPK) signaling, protein kinase R (PKR) signaling, and PKC signaling [129,130,131,132]. Resveratrol is a promising food additive for DR patients, as it was also shown to inhibit endothelial-to-mesenchymal transition and decrease retinal vascular permeability [129,131].

#### 4.3.3. Plant Polyphenol Extracts

- Lychee seed polyphenol

Litchi seeds, rich in diverse bioactive compounds like flavonoids and saponins, are traditionally used in some Chinese medicine formulas with the efficacy of promoting qi and dispelling stagnation [133,134]. A purified fraction containing polyphenols in *Lychee seed* (LSP) was recently proven to ameliorate DR by suppressing HG-induced inflammation and apoptosis and hindering angiogenesis [133,135]. These results expanded the therapeutic potential of LSP and helped to extend the usage of these ostensibly obsolete seeds.

- Sophora flavescens Aiton extract (SFE)

*Sophora flavescens* Aiton or “Kushen” is a widely used traditional Chinese medicine. A recent study based on untargeted retinal metabolomics explored the distribution of flavonoids of SFE in rat’s aqueous humor and retina after oral administration of SFE [136]. Notably, SFE was reported to guard against DR by regulating the synthetic metabolic pathways, enhancing the adoption of SFE in clinical practice [136].

- Hawthorn polyphenol extract (HPE)

Hawthorn contains various bioactive compounds including flavonoids and triterpenoids with pharmacological benefits for different diseases [137]. It was demonstrated that HPE inhibited oxidative damage, inflammation, and apoptosis via the AMP-activated protein kinase (AMPK)/sirtuin 1(SIRT1)/NF-κB pathway and miR-34a/SIRT1/p53 pathway in ARPE-19 retinal pigment epithelial cells [138]. This study supported the use of HPE as a food supplementation for attenuating hyperglycemia-induced retinal damage [138].

- Homoisoflavonoids from the Hyacinthaceae (*sensu APGII*)

Hyacinthus subfamily members hold an important place in traditional medicine. The potent homoisoflavonoids from the Hyacinthaceae (*sensu APGII*) displayed anti-proliferative activity and in vitro anti-angiogenic efficacy in HRECs [139].

### 4.4. Saponins

Saponins are a large group of amphiphilic glycosides of steroids and triterpenes present mainly in land plants. Ginsenosides, a specific class of saponins, are the major pharmacologically active components of ginseng plants like *Panax ginseng* [140]. There are various specific types of ginsenosides including ginsenosides Rg1 (GRg1), ginsenosides Rb1 (GRb1), ginsenosides Rd (GRd), and ginsenosides Re (GRe). GRg1 has received great interest over the years. GRg1 was reported to inhibit proliferation, migration, and angiogenesis in the HG-insulted HREC via the lncRNA SNHG7/miR-2116-5p/sirtuin-3 (SIRT3) axis [141]. Peroral supplementation of GRg1 alleviated inflammation, inhibited angiogenesis, and decreased cell apoptosis in retinal neurons in the genetically diabetic (db/db) mice and STZ-induced DR rats [142,143]. GRg1 can also be administered as eye drops to mice with DR. After 15 days of eye drop administration, the db/db mice yielded better visual function [144]. Other ginsenosides, GRe, GRd, and GRb1 also showed strong potential to scavenge ROS and inhibit apoptosis and angiogenesis, attenuating HG-induced cell injury [145,146,147,148].

Notoginsenoside R1 (NGR1) is a distinctive bioactive saponin compound found mainly in the roots of *Panax notoginseng*, another well-known medicinal herb. Oral NGR1 was shown to rescue retinal function by inhibiting mitophagy and apoptosis, alleviating oxidative stress and inflammation through PTEN-induced putative kinase protein 1 (PINK1)-dependent activation of mitophagy in db/db mice [149]. A recent study revealed that *Panax notoginseng* saponins, which contain NGR1, GRg1, GRe, GRb1, and GRd, could alleviate DR by inhibiting retinal inflammation via the NF-κB signaling pathway with the assumption that GRg1 and GRb1 may be the major pharmacological components [150]. These studies indicate that saponins carry the potency to alleviate DR, but more in vivo studies and clinical trials are still warranted.

**Table 1:** Effects of nutraceuticals in DR-related models (basic studies).

Dietary Source/Compound	In Vivo/In Vitro	Dosage	Duration	Cell Culture/Animal Model	Effects	Year	Refs.
Oil							
DHA for cells; ω3-PUFAs for mouse	In vitro and in vivo	DHA (50 μM)	24 h	DMOG treated HRMECs	AMPK-dependent anti-neovascularization effect	2023	[63]
		50 μL of oil rich in ω3-PUFAs (240 mg/g/day of DHA and 360 mg/g/day of EPA), (p.o.)	P1–P17 for nursing mother	Mouse OIR model			
Carotenoids							
Astaxanthin	In vitro	10, 20, 40 μM	Pretreated for 6 h	H_2_O_2_-induced and UVB-induced oxidative stress in ARPE-19 cells	Antioxidative effect	2022	[83]
Astaxanthin	In vitro	10, 20, 50 μM	24 h	661 W cell line	PI3K/Akt/Nrf2 pathway mediated antioxidative and anti-apoptotic effects	2022	[82]
Lutein	In vitro	0.5, 1.0 μM	24 h	HG-induced damage in ARPE-19 cells	PI3K/AKT/Nrf2 and Erk1/2 pathways mediated antioxidative effect; protected RPE from diabetes-associated damages	2020	[78]
Astaxanthin	In vivo	10 or 100 ng/µL (i.vit.); 0.5 or 5 mg/kg (i.p.)	Once	OIR (C57BL/6J mice)	Anit-neovascularization and anti-apoptotic effects	2019	[84]
Astaxanthin	In vivo	10 or 20 mg/kg/day (p.o.)	21 days	STZ-induced DR (Swiss albino mice)	Inhibited processes of neuron-specific enolase activity; improved visual acuity function; controlled blood glucose; regulated oxidative stress (catalase) and neuron-specific enolase activity	2022	[85]
Astaxanthin	In vivo	20 mg/kg/day (p.o.)	45 days	STZ-induced DR (rats)	Nrf2/keap1 pathway mediated anti-apoptotic, anti-inflammation, and antioxidative effects	2023	[88]
Astaxanthin	In vivo	4.8 mg/kg/day (p.o.)	7 days	Hyper caloric diet-induced DR (*Psammomys obesus*)	Inhibited aldose reductase activity	2019	[87]
Carotenoid-enriched carrot powder diet	In vivo	β-carotene (61.9 mg/kg/day), α-carotene (43.1 mg/kg/day) (p.o.)	9 weeks	STZ-induced DR (rats)	Exacerbated retina dysfunction	2020	[94]
Lutein	In vivo	Water dispersible form of lutein (4.2 or 8.4 mg/kg/day) (p.o.)	From 6 weeks to 9 months	Ins2^Akita/+^ mice	Suppressed retinal inflammation, protected retinal vasculature, and preserved retinal function	2020	[77]
Lutein or lactucaxanthin	In vivo	Lutein or lactucaxanthin micelles (200 μM, i.e., 0.23 mg/kg/day body weight of rats) (p.o.)	8 weeks	STZ-induced DR (rats)	Downregulated ER stress (ATF4, ATF6, and XBP1), inflammatory markers (TNF-α, IL-6, NF-κB, and ICAM-1), and VEGFA. Antioxidative, anti-inflammatory effects	2023	[90]
Lutein-rich purple sweet potato leaf extract	In vivo	200 or 400 mg/kg/day (p.o.)	12 weeks	STZ-induced DR (rats)	Suppressed retinal inflammation, increased retina thickness	2023	[92]
Lutein and trapa bispinosa roxb. extract (TBE)	In vivo	Solidified food mixed with lutein (13.3 mg/kg/day) and TBE (133.3 mg/kg/day)	8 weeks	C57BL/ksj-db/db mice	Downregulated GFAP and VEGF; improved the impaired regulation of retinal blood flow	2022	[151]
Lycopene	In vivo	4 mg/kg/day (p.o.)	3 months	Alloxan-induced diabetic optic neuropathy	Antioxidative, anti-inflammatory, ameliorated the tissue damage on the optic nerve	2018	[81]
Palm oil mill effluent-derived beta-carotene	In vivo	50 or 100 mg/kg/day (p.o.)	21 days	STZ-induced DR (rats)	Regulated BRB function; antioxidative effect	2023	[93]
Lutein	In vitro and in vivo	Lutein (10 µM) or oxidized lutein (40 nM)	48 h	HG-induced damage in ARPE-19 cells	Activated AMPK and quenched ROS to maintain mtDNA integrity and mitochondrial biogenesis; antioxidative, anti-inflammatory effects	2019	[79]
		One dose/day (p.o.)	8 weeks	STZ-induced DR (rats)			
Lutein or lactucaxanthin	In vitro and in vivo	Lutein (10 μM) or lactucaxanthin (5 or 10 μM)	48 h	HG-induced damage in ARPE-19 cells	Downregulated angiogenesis markers through HIF-1α/ER stress/VEGF axis; antioxidative and anti-angiogenic effects, altered the retina structural damages	2021	[91]
		Lutein or lactucaxanthin micelles (200 μM, i.e., 0.23 mg/kg/day) (p.o.)	8 weeks	STZ-induced DR (rats)			
Astaxanthin	In vivo and in vitro	1, 5, 10 μM	48 h	HG-induced damage in ARPE-19 cells	Antioxidative and anti-inflammatory effects through PI3K/Akt/NF-κB pathway	2022	[86]
		Micellar astaxanthin (3 mg/kg/day) (p.o.)	8 weeks	STZ-induced DR (rat)			
Astaxanthin	In vivo and in vitro	1, 5, 10 μM	48 h	ARPE-19 cells under hyperglycemic w/o hypoxic (CoCl_2_) condition	Downregulated VEGF through HIF-1α and XBP1 signaling pathway; restored ZO-1; modulated the diabetes-induced retinal morphological changes	2021	[89]
		Micellar astaxanthin (3 mg/kg/day) (p.o.)	8 weeks	STZ-induced DR (rats)			
Polyphenol							
Anthocyanin	In vitro	Cyanidin-3-O glucoside (10 μM)	Pretreated 1 h and incubated for 24 h	HG-induced damage in ARPE-19 cells	Antioxidative effect	2022	[104]
Anthocyanin	In vitro	Blueberry anthocyanin extracts (12.5, 25, and 50 mg/L); Cyanidin-3-O glucoside (5, 10, 20 μM)	12 h, 24 h, or 48 h	HG-induced injury in ARPE-19 cells	Antioxidative effect mediated by REDD1/GSK3β pathway; downregulated VEGFA	2023	[106]
Anthocyanin; Verbascoside	In vitro	Cyanidin-3-O glucoside (5, 10, or 50 μM), Verbascoside (5, 10 or 50 μM)	48 h	HG-induced damage in HRECs	Antioxidative effect; protected integrity (ZO-1, VE-cadherin ↑) and function of epithelial cell layers	2022	[107]
Catechin 7-O-β-D-apiofuranoside (C7A)	In vitro	4 μg/mL	72 h	HG-induced injury in HRMECs and pericytes	Inhibited pericyte apoptosis by reducing p38 and JNK activity; reduced endothelial permeability	2023	[121]
Curcumin	In vitro	10 µM	12 h	HG-induced injury in ARPE-19	Anti-inflammatory effect via ROS/PI3K/AKT/mTOR signaling pathway	2019	[126]
Curcumin	In vitro	15 µM	24 h	HG-induced injury in ARPE-19	Antioxidative effect via ERK1/2-mediated Nrf2/HO-1 pathway	2019	[125]
Epigallocatechin-3-gallate (EGCG)	In vitro	10, 20, or 30 μM	24 h	HG-induced damage in rat primary retinal Müller cells	Stimulated autophagy and reduced apoptosis	2019	[119]
Hawthorn polyphenol extract (HPE)	In vitro	10 μg/mL	24 h	HG-induced injury in ARPE-19	Regulated mir-34a/SIRT1/p53 signaling to reduce acetylation; inhibited oxidative damage, inflammation, and apoptosis through AMPK/SIRT1/NF-κB pathway	2021	[138]
Homoisoflavonoids from the Hyacinthaceae (*sensu* APGII)	In vitro	0.001–0.5 µM	48 h	HRECs	In vitro anti-angiogenic efficacy and anti-proliferative activity	2019	[139]
Naringenin	In vitro	1 or 10 µM	24, 48, and 72 h	HG-induced injury in HRECs	Antioxidative and anti-apoptotic effects	2022	[122]
Peracetate-protected epigallocatechin-3-gallate (pro-EGCG)	In vitro	10, 20, 30 μM	24 h	HG-induced damage in mouse primary retinal Müller cells	Anti-inflammatory effect via inhibition of ROS/TXNIP/NLRP3 inflammasome; mitigated cell proliferation and pro-angiogenic factor production	2020	[120]
Quercetin	In vitro	20, 40, or 80 µM	48 h	HG-induced injury in HRMECs	Inhibited NLRP3 inflammasome-mediated inflammation and autophagy, inhibited angiogenesis	2021	[112]
Resveratrol	In vitro	1 µM	24 h	HG-induced injury in HRECs	Inhibited PKC, antioxidative effects; inhibited NOX-mediated endothelial-to-mesenchymal transition	2021	[131]
Curcumin	In vivo	200 mg/kg/day (p.o.) (combined with subcutaneous insulin, 4–6 IU/day)	4 weeks	STZ-induced DR (rats)	Nrf2/HO-1 pathway mediated antioxidative and anti-apoptosis effects; improved retina morphology	2021	[124]
Naringenin	In vivo	25, 50, or 75 µM	3 h post-fertilization to 5 days post-fertilization	HG-induced DR (zebrafish)	Antioxidative effect; regressed the glucose levels and cellular damage; inhibited of macular degeneration	2023	[123]
Quercetin	In vivo	50 mg/kg/day (p.o.)	12 weeks	HFD and STZ-induced DR (rat)	Downregulated levels of blood glucose and oxidative stress, inhibited inflammation and improved dysbacteriosis and retinal function through gut–retina axis and Nrf2 pathway	2024	[116]
Quercetin	In vivo	150 mg/kg/day (p.o.)	16 weeks	STZ-induced DR (rats)	Anti-inflammatory, anti-angiogenic, and neurotrophic effects by inducing HO-1	2021	[113]
Quercetin	In vivo	35, 70 mg/kg (p.o.)	12 weeks	db/db mice	Inhibited oxidative stress, apoptosis, and neurodegeneration via SIRT1/ER signaling pathway	2020	[114]
Resveratrol (*trans*-resveratrol)	In vivo	5 mg/kg (i.p.)	Five days a week for one month	STZ-induced DR (rats)	Anti-apoptotic effect through MAPK signaling (CASP3, JNK1, p38αMAPK, ERK1 ↓; Bcl-2 ↑)	2021	[132]
Resveratrol	In vivo	5 and 10 mg/kg/d (p.o.)	30, 32, 34, and 36 weeks	STZ-induced DR (rats)	RAX/P-PKR pathway-mediated anti-apoptotic effects	2022	[130]
Rutin	In vivo	50 mg/kg (p.o.)	24 weeks	STZ-induced DR (rats)	Downregulated the levels of VEGF, TNF-α, and aldose reductase; antioxidative effect; reduced retinal vessel tortuosity index	2019	[117]
*Sophora flavescens* Aiton extract	In vivo	37.5, 75, 150 mg/kg/day (p.o.)	4 months	HFD and STZ-induced DR (rats)	Antioxidative, anti-inflammatory effects; ameliorated retinal morphological changes	2022	[136]
Anthocyanin	In vitro and in vivo	150, 250, or 450 μg/mL	Pretreated 2 h and incubated for 48 h for cells	HG-induced human RPECs injury	Anti-apoptotic and antioxidative effects; alleviated ER stress; improved retina morphology changes	2023	[105]
		35 or 140 mg/kg/day (p.o.)	6 weeks	STZ and high fat and high sucrose diet-induced DR rats			
Anthocyanin (Cyanidin-3-O glucoside)	In vitro and in vivo	10 µM	48 h	HG/CoCl_2_-induced damage in HRECs, BV2 microglia cells	Alleviated the inflammation, microglial activation, and angiogenesis; inhibited vascular leakage	2021	[108]
		20 mg/kg/day (p.o.)	1 month	STZ-induced DR (mice)			
Lychee seed polyphenol	In vitro and in vivo	8, 16, or 32 μg/mL	/	HG-induced damage in HRECs	Inhibited NLRP3 inflammasome-related inflammation; upregulated tight junction proteins; anti-angiogenesis and anti-apoptotic effects	2023	[133]
		50, 100, or 200 mg/kg/day (p.o.)	6 weeks	db/db mice			
Resveratrol	In vitro and in vivo	10, 50, or 100 µM	24 h	HG-induced injury in rat retinal endothelial cells	Decreased retinal vascular permeability, inhibited retinal apoptosis, anti-inflammatory effect	2019	[129]
		0.1 or 1 μg/mL (i.vit.); 5, 10, or 50 μg/kg/day (tail vein injection)	12 weeks	STZ-induced DR (rats)			
Quercetin (Q) and its 8-methyl pentamethyl ether derivative (8MQPM)	In vitro and ex vivo	25 µM	24 h	HRECs treated with conditioned medium from Y-79 human retinoblastoma cell line or stimulated by VEGFA	Akt, ERK, and JNK mediated anti-angiogenic effect	2019	[115]
		/	/	Ex vivo rabbit aortic ring assay			
Saponins							
Ginsenoside Rb1 (GRb1)	In vitro	5, 10, 20 μM	48 or 72 h	HG-induced injury in rat retinal capillary endothelial cells	Quenched ROS and inhibited DNA damage and apoptosis through NMNAT–NAD–PARP–SIRT axis	2019	[146]
Ginsenoside Re (Re)	In vitro	3 μM	24 h	HG-induced retinal endothelial RF/6A cell injury	Anti-angiogenesis and anti-apoptotic effects through upregulating PI3K/AKT pathway and inhibiting HIF-1α/VEGF signaling	2020	[148]
Ginsenoside-Rg1 (GRg1)	In vitro	10 μM	48 h	HG-induced injury in HRECs	Inhibited HG-induced proliferation, migration, and angiogenesis via lncRNA SNHG7/mir-2116-5p/SIRT3 axis	2022	[141]
Ginsenoside Rb1 (GRb1)	In vivo	20, 40 mg/kg/day (i.p.)	4 weeks	STZ-induced DR (rats)	Antioxidative effects	2019	[147]
Ginsenoside-Rg1 (GRg1)	In vivo	25, 50, or 225 mg/kg/day (p.o.)	8 weeks	db/db mice	Inhibited GCL and INL cell apoptosis	2020	[142]
Ginsenoside-Rg1 (GRg1)	In vivo	2.5, 5, or 10 mg/kg (eye drops, twice a day)	15 days	C57BL/ksj-db/db mice	IRS-1/Akt/GSK3β signaling-mediated neuroprotective effect	2019	[144]
Ginsenoside Rd (GRd)	In vitro and in vivo	1, 3, 10, or 30 μΜ	24 h	HG-induced injury in HUVECs	Downregulated NOX2 and ameliorated oxidative stress, mitochondrial dysfunction, and endothelial apoptosis via AMPK/SIRT1 signaling	2022	[145]
		10 μL 1% (*w*/*v*) GRd as eye drops, daily	1 month	STZ-induced DR (C57BL/6 mice)			
Ginsenoside-Rg1 (GRg1)	In vitro and in vivo	10 μM	48 h	HG-induced injury in HRMECs	Anti-angiogenesis and anti-inflammatory effects via mir-216a-5p-mediated downregulation of TLR4/NF-κB signaling pathway	2023	[143]
		0.5 mL (5 g/mL) (p.o.)	16 weeks	STZ-induced DR (rats)			
*Panax notoginseng* saponins	In vitro and in vivo	25 μM	24 h	HG-induced injury in MIO-M1 cells	NF-κB signaling pathway-mediated anti-inflammatory effect; attenuated elevated BRB disruption	2024	[150]
		40, 80, or 160 mg/kg (p.o.)	1 month	STZ-induced DR (rats)			
Notoginsenoside R1 (NGR1)	In vitro and in vivo	5, 10, 20, 40 μΜ	72 h	HG-induced injury in rMC-1 cells	Enhanced PINK1-dependent activation of mitophagy; downregulated VEGF and upregulated PEDF; inhibited apoptosis; antioxidative and anti-inflammatory effects; restored retinal function	2019	[149]
		30 mg/kg/day (p.o.)	12 weeks	db/db mice			

Abbreviations: Administration routes: i.p., intraperitoneal injection; i.vit., intravitreal injection; p.o., per os (by mouth). Others: AMPK, AMP-activated protein kinase; ATF4, activating transcription factor 4; ATF6, activating transcription factor 6; BRB, blood–retinal barrier; CASP3, caspase-3; DHA, docosahexaenoic acid; DMOG, dimethyloxalylglycine; ER, endoplasmic reticulum; Erk1/2, extracellular signal-regulated kinase 1/2; GCL, ganglion cell layer; GFAP, glial fibrillary acidic protein; GSK3β, glycogen synthase kinase 3 beta; HG, high glucose; HFD, high-fat diet; HIF-1α, hypoxia-inducible factor-1 alpha; HRECs, human retinal endothelial cells; HRMECs, human retinal microvascular endothelial cells; HUVECs, human umbilical vein endothelial cells; ICAM-1, intercellular adhesion molecule-1; INL, inner nuclear layer; IRS-1, insulin receptor substrate 1; JNK, c-Jun N-terminal kinase; keap1, Kelch-like ECH-associated protein 1; NAD, nicotinamide adenine dinucleotide; NF-κB, nuclear factor-kappa B; NLRP3, NOD-like receptor family, pyrin domain-containing 3; NMNAT, nicotinamide mononucleotide adenylyltransferase; NOX, NADPH oxidase; Nrf2, nuclear factor erythroid 2-related factor 2; OIR, oxygen-induced retinopathy; PARP, poly(ADP-ribose) polymerase; PEDF, pigment epithelium-derived factor; PI3K, phosphoinositide 3-kinase; PKC, protein kinase C; RAX/P-PKR pathway, retinal apoptosis through RAX/PKR pathway; REDD1, regulated in development and DNA damage response 1; RPEC, retinal pigment epithelial cells; ROS, reactive oxygen species; SIRT1, sirtuin 1; SNHG7, small nucleolar RNA host gene 7; STZ, streptozotocin; TLR4, toll-like receptor 4; TNF-α, tumor necrosis factor-alpha; TXNIP, thioredoxin-interacting protein; UVB, ultraviolet B; VEGF, vascular endothelial growth factor; VEGFA, vascular endothelial growth factor A; VE-cadherin, vascular endothelial cadherin; XBP1, X-box binding protein 1; ZO-1, zonula occludens-1; ↓: down-regulated; ↑: up-regulated.

**Table 2:** Effects of nutraceuticals in DR (clinical studies).

Dietary Compound	Family of Compound	Type	Dosage	Supplementation Period	Candidates	Effects	Year	Refs.
Omega-3 PUFAs (DHA/EPA)	Oil	Prospective	1 g omega-3 fatty acids (containing 460 mg EPA and 380 mg DHA) daily	6.5 years	T1DM/T2DM with or without DR	No benefits for DR.	2023	[69]
DHA	Oil	Prospective	1050 mg/day	2 years	NPDR (any stage)	Did not appear to influence the slowing of the progression of NPDR	2022	[68]
Brudyretina: high rich DHA (1050 mg/d) nutraceutical formulation, (DHA, EPA, a mixture of B vitamins, vitamins C and E, lutein, zeaxanthin, and minerals)	Oil plus others	Prospective	3 capsules of Brudyretina 1.5 g once daily	90 days	T2DM with NPDR (24 patients)	No difference in BCVA and central subfield macular thickness. Total antioxidant capacity levels increased, and plasma IL-6 levels decreased	2018	[152]
Lutein	Carotenoids	Prospective	10 mg/day of lutein	36 weeks	T2DM with NPDR (30 patients)	Improved contrast sensitivity	2017	[95]
Lutein (L) and zeaxanthin (Z)	Carotenoids	Retrospective	Z (0.5 mg/day) or Z (0.5 mg/day) + L (6 mg/day)	4 months	NPDR (72 patients)	Lutein supplement showed no benefits	2019	[97]
Lycopene	Carotenoids	Cross-sectional	Lycopene intake was assessed by using a food frequency questionnaire	/	T2DM patients and healthy controls	Ameliorated oxidative stress	2021	[96]
Anthocyanin	Polyphenol	Prospective	320 mg/day	4 weeks	Healthy controls; T2DM; T2DM-at-risk	Anti-inflammatory (IL-6, IL-18, TNF-α ↓)	2021	[110]
Curcumin, artemisia, bromelain, and black pepper	Combo	Prospective	Curcumin (200 mg), artemisia (80 mg), bromelain (80 mg), and black pepper (2 mg)	6 months	T2DM with or without mild to moderate DME	Increased central retinal thickness, visual acuity, and vessel density of the deep capillary plexus	2022	[127]

Abbreviations: BCVA, best-corrected visual acuity; DHA, docosahexaenoic acid; DME, diabetic macular edema; DR, diabetic retinopathy; EPA, eicosapentaenoic acid; IL-6, interleukin-6; IL-18, interleukin-18; NPDR, non-proliferative diabetic retinopathy; PUFA, polyunsaturated fatty acids; T1DM, type 1 diabetes mellitus; T2DM, type 2 diabetes mellitus; TNF-α, tumor necrosis factor-alpha; ↓: down-regulated.

## 5. Delivery Systems

Despite the powerful health-promoting properties of nutraceuticals such as antioxidant and anti-inflammatory properties, the bioavailability of most nutraceuticals is low. The oral bioavailability of nutraceuticals mainly involves the following processes: liberation from the food structures, solubilization in the digestive fluids, interaction with gastrointestinal components, epithelial cell permeation, chemical disintegration, and metabolic transformation [153,154]. Carotenoids are known to be lipophilic and extremely vulnerable to environmental stresses like heat, oxygen, light exposure, and unfavorable pH in the gastrointestinal tract (GIT). The tendency towards oxidative degradation, incomplete release from the food matrix, and low solubility have resulted in their poor bioavailability [155,156]. As for polyphenols, despite the abundance of studies investigating the effects of polyphenols on human health, only a few studies have considered the bioavailability of polyphenols to further support their bio-effect in vivo. The bioavailability varies among different classes of polyphenols; but, in general, polyphenols have a low oral bioavailability, which is mainly due to extensive biotransformation mediated by phase I and phase II reactions in the enterocytes and liver, as well as in the intestinal microbiota [157,158].

**Figure 2:**
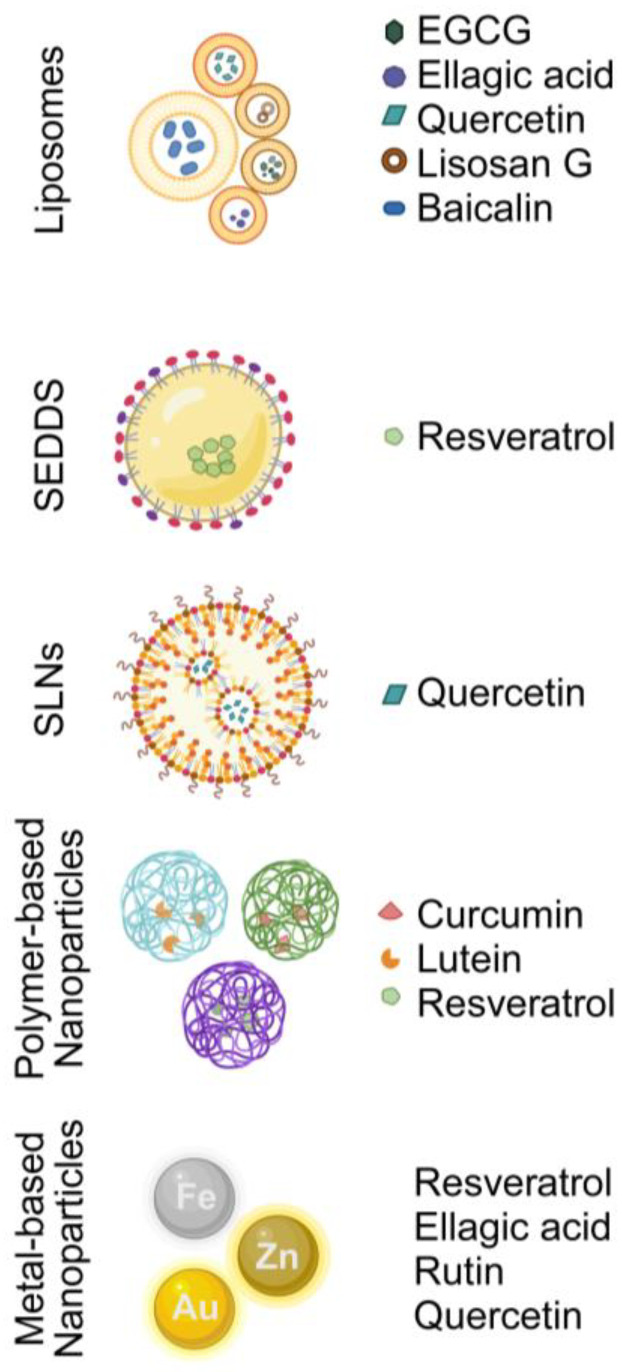
An illustration of the delivery systems mentioned in the present review. (Abbreviations: EGCG, epigallocatechin gallate; SEDDS, self-emulsifying drug delivery system; SLNS, solid lipid nanoparticles).

Furthermore, the blood–ocular barrier hinders the entry of peroral bioactive compounds into the posterior segment. Over the decades, scientists have attempted to introduce and refine nutrient carriers and delivery systems to yield higher bioavailability and, thus, better efficacy. Nanoencapsulation technology has been developed to overcome unwanted conditions such as gastrointestinal digestion and cellular metabolism. Here, we discussed a few of the typically used classes of nano-delivery systems and summarized some nutraceutical-loaded nanocarriers that can be potentially applied in DR (Figure 2 and Table 3). The classification of nanocarriers could fall into three groups: (i) lipid and surfactant-based nanocarriers, (ii) biopolymer-based nanocarriers, and (iii) metal-based nanoparticles [154,159].

### 5.1. Lipid and Surfactant-Based Nanocarriers

#### 5.1.1. Nanoemulsions

Nanoemulsions (NEs) are emulsion systems carrying droplets with diameters ranging between 50 and 1000 nm. They exist in multiple forms including oil-in-water (O/W) NEs and water-in-oil (W/O) NEs [160]. It is well-demonstrated that carotenoids can be absorbed more easily when administered together with fat [161]. Olive oil, rich in oleic acids, was reported to boost the intestinal absorption of lutein in mice [162]. Using NE technology, lutein-encapsulated oleic–linoleic acid O/W NEs could improve the oral bioavailability of lutein (5.83 folds bioaccessibility of free lutein) in rats [163]. Furthermore, smaller O/W NEs containing lutein (~10 nm) are promising candidates for topical ocular application to eye diseases related to oxidative stress [164]. It was demonstrated that with penetratin modification, lutein NEs could not only be better taken up by ARPE-19 retinal pigment epithelial cells but also show higher bioactivity in inhibiting HUVEC migration [165]. In addition, with the assistance of an in situ gel, the residence time of the pre-corneal drug containing lutein in the eye was prolonged when instilled in the lower conjunctival sac in the rats; this further increased the amount of drug reaching the fundus, alleviating the sodium iodate-induced retina damage [165]. Despite the better stability and bioavailability, these lutein NEs have not been tested in in vitro or in vivo models of DR, and extended investigation is needed to promote the applications of NEs in DR. Apart from lutein, it is also worthwhile to develop and explore NE technology for other carotenoids in the context of DR.

#### 5.1.2. Liposomes and Nanoliposomes

Liposomes are spherical or oval vesicles with phospholipid bilayers and an aqueous core with diameters ranging from tens of nanometers to hundreds of micrometers. Nanoliposomes are nano-sized liposomes with diameters below 200 nm [166]. Liposomes have been wildly applied in the drug industry for their ability to carry and enhance the stability of hydrophilic (in the aqueous core) and lipophilic (between the lipid bilayers) substances [166,167]. In addition, the phospholipid bilayers in liposomes allow for high biocompatibility; when modified with targeting ligand-like antibodies, targeted delivery can be achieved [168].

The application of liposomes in encapsulating certain nutraceuticals has been investigated over recent years. Baicalin is a flavonoid with anti-inflammatory, antioxidant, and anti-angiogenic effects in the eye [169,170]. However, baicalin is poorly soluble and unstable in basic pH. In order to improve its bioavailability, Ashraf et al. fabricated and compared different baicalin vesicular systems (liposomes, penetration enhancer vesicles, and transfersomes) and found that baicalin liposomes displayed the highest absorption rate [171]. Another example is anthocyanins, which are unstable and sensitive to many influences, such as oxygen, temperature, and UV radiation. Anthocyanins-loaded nanoliposomes manufactured with ultrasonication showed higher stability, cellular uptake by Caco-2 cells, and antioxidative properties in vitro [172]. It was reported that N-trimethyl chitosan (TMC)-coated liposomes of C3G (a specific type of anthocyanins) could alleviate the oxidative stress caused by selenite sodium in rats as a precorneal drug [173]. Likewise, TMC-coated flexible liposomes with resveratrol were shown to pass through the biological barrier and reach the ocular fundus as eye drops to mitigate the blue-light-induced retinal damage in ICR mice [174].

For diabetic complications, the application of liposomes has also been explored. Oral administration of pegylated quercetin liposomes elevated quercetin concentrations in plasma compared with non-encapsulated quercetin, resulting in significant attenuation of the oxidative stress indicators in serum and kidneys and amelioration STZ-induced diabetic nephropathy in rats [175]. Similarly, intraperitoneal injection of liposomal EGCG was demonstrated to suppress oxidative stress and enhance the total antioxidant capacity of plasma (TAC), thiols, and catalase in STZ-induced DR rats [176]. In addition, EGCG-loaded nanoliposomes could lower the level of matrix-metalloproteinase-2 (MMP-2) and -9 (MMP-9), which are important in the progression of DR [176]. Lisosan G (LG) is a fermented powder acquired from whole grains that is protective of DR due to its abundance of bioactive substances, like polyphenols, alpha-lipoic acid, PUFAs, and vitamins [177]. Recently, a 6-week gavage of LG-encapsulated liposomes was shown to significantly rescue the retinal function of STZ-induced DR [178].

Besides encapsulating nutraceuticals in liposomes, specific ligands can be added to modify the liposomes, such as the isoDGR, a tripeptide motif that can combine with αvβ3 integrin [179]. Li and his colleagues invented one kind of liposome co-loaded with ellagic acid and hemoglobin and adjusted with TAT peptide to promote cellular uptake as well as isoDGR to achieve targeted delivery (EA-Hb/TAT&isoDGR-Lipo) [180]. EA-Hb/TAT&isoDGR-Lipo displayed great efficacy when administered intravenously or as an eye drop in ameliorating DR in db/db mice by scavenging ROS, downregulating the expression of GFAP, HIF-1α, VEGF, and p-VEGFR2 [180].

#### 5.1.3. Self-Emulsifying Drug Delivery Systems (SEDDSs)

Different ratios of oils and surfactants, together with lipophilic substances, form isotropic solutions called SEDDSs. SEDDSs are named after their spontaneous formation of emulsions or microemulsions after being added to water and lightly agitated [154]. With droplet sizes smaller than 200 nm, self-nanoemulsifying drug delivery systems (SNEDDSs) are superior for their good absorption rate and dispersion in the tissue [181]. Zingale et al. developed an SNEDDS loaded with resveratrol (RSV-SNEDDS) and tested its biocompatibility in a rabbit corneal epithelial cell line (SIRC) [182]. The RSV-SNEDDS displayed improved solubility, stability, and bioavailability, suggesting that SNEDDSs can be used as promising nanocarriers for drugs to deliver toward the posterior ocular segment [182].

#### 5.1.4. Solid Lipid Nanoparticles

Solid lipid nanoparticles (SLNs), consisting of solid lipids with melting points higher than 40 °C, are a nontoxic nanocarrier system that provide outstanding solubilization of lipophilic compounds and excellent protection of the contents in the complex environment of the gastrointestinal tract [154]. SLNs of quercetin, a nanoformulation of quercetin (NQ), were generated and demonstrated competence in treating STZ-induced DR in a zebrafish model [183]. Intraperitoneal injection of NQ restored the retinal function in the STZ-induced DR zebrafish by lowering the blood glucose level, regulating the homocysteine pathway, and scavenging ROS [183,184].

### 5.2. Polymer-Based Nanocarriers

Apart from the lipid-based and surfactant-based nanocarriers, biopolymeric nanoparticles using biopolymer-like polysaccharides, proteins, polyethylene glycol (PEG), and polylactic acid are another important category of nano-delivery system [154]. Toragall and Baskaran developed a lutein-loaded chitosan–sodium alginate-based nanocarrier system (LNCs) and found that LNCs led to higher retinal lutein levels in STZ-induced DR rats when administered orally, with 2.80-fold of area under the curve (AUC) compared to the micellar lutein-treated group [185]. Later, they found that LNCs could slow and control the release of lutein from LNCs, and showed better cellular antioxidant and anti-angiogenic efficacy in ARPE-19 retinal pigment epithelial cells by activating Nrf2 translocation and downregulating the level of VEGF through HIF-1α and activating transcription factor-4 (ATF4)/X-box binding protein 1 (XBP-1) signaling [186].

Resveratrol has also been reported to be modified and loaded in a polyelectrolyte microcapsule and covered by rhodamine 6G (PMs-Rv-Rh6G) [187]. PMs-Rv-Rh6G exhibited a high ratio (60%) of internalization by D407 cells with anti-VEGF and anti-inflammatory effects, suggesting its potential application as an intravitreal injection to treat DR [187]. Besides being used in management alone, nanoparticles of nutraceuticals used in combination with the standard treatments can achieve better outcomes. It was demonstrated that the STZ-induced DR rats treated with insulin jointly with oral delivery of the curcumin-laden double-headed nanoparticles displayed ameliorated diabetic cataracts and retinopathy compared to those treated with insulin alone [188].

### 5.3. Metal-Based Nanoparticles

Comprised of metals or metal oxides, metal-based nanoparticles enjoy a promising future in drug or gene delivery and magnetic diagnostics as they can be conjugated with antibodies, ligands, and contents of interest [189]. Marella et al. used ZnO nanoparticles to encapsulate ellagic acid (NEA) [190]. The NEA showed improved solubility and biological inhibitory actions on aldose reductase and α-glucosidase but at the same time displayed minimal toxicity and a low degradation rate of ellagic acid [190]. Gui et al. produced an ultrasmall (5–10 nm) coordination polymer nanodots nanozyme by coupling quercetin with low-toxic iron ions (Fe-Quer NZs) [191]. It was reported that Fe-Quer NZs mimicked the activity of three antioxidant enzymes, superoxide dismutase, peroxidase, and catalase, and, in turn, showed an excellent ability to protect against inflammation, oxidative stress, microvascular leakage, and angiogenesis in STZ-induced diabetic rats [191]. Moreover, data from the optical coherence tomography angiography (OCTA) imaging reveal that oral intake of Fe-Quer NZs restored the retinal blood flow and fundus blood density in STZ-damaged retinae, suggesting their utilization in early NPDR [191].

Gold nanoparticles (AuNPs) are highly flavored as drug delivery systems for their chemical stability, low toxicity, easy functionalization (strong affinity for functional groups like thiols), and photo-responsive activity [192]. When rutin was conjugated with AuNPs, these nanoparticles (AuNPsR) showed higher oral bioavailability with increased antioxidant capacity and the function to improve the fundus appearance of retinal arterioles [193]. Stoia et al. utilized the strengths of the active anisotropic gold bipyramidal nanoparticles (AuBPs) as efficient photothermal agents and fabricated a resveratrol-encapsulated nanocarrier with a calcium carbonate core (CaCO_3_ MC-AuBPs) [194]. CaCO_3_ MC-AuBPs are of great interest in treating retinal diseases like DR as they process the power of the controlled release of therapeutic resveratrol based on the laser-induced thermoplasmonic effect at the retinal level [194].

Interestingly, bioactive nutraceuticals can not only be the core compounds loaded in the nanoparticles, but they can also serve as green adjunctive reagents in the process of synthesis of drug delivery systems [195]. For example, resveratrol was used as a stabilizing and reducing agent when fabricating a type of AuNPs [196]. These resveratrol-coated AuNPs demonstrated a remedial effect on STZ-induced DR in rats [196]. Such manifestations all highlight the great potential of nutraceuticals in DR.

**Table 3:** A summary on the studies in delivery systems for nutraceuticals in DR-related models.

Compound	In Vivo or In Vitro	Dosage and Administration Way	Name of the Product	Particle Size (nm)	Cell Culture/Animal Model	Effects	Improvements	Year	Refs.
Nanoemulsions									
Lutein	In vivo	600 μM of lutein (p.o.)	Lutein-NEL	110 ± 8	Rats	/	Improve the bioaccessibility of lutein	2021	[163]
Liposomes									
Baicalin	In vivo	As eye drops, instilled (100 μL) in the conjunctival sac	Baicalin vesicles	667–1341	Rabbits	Antioxidative	Improve stabilization, sterilization endurance, and safety with pharmacokinetic superiority	2018	[171]
Epigallocatechin-5-gallate (EGCG); liposomal EGCG	In vivo	2.5 mg/100 g b.w./day (i.p., once a day for two consecutive days before STZ administration)	Liposomal nanoformulation of EGCG	170	STZ-induced DR (rats)	Antioxidative	Superior antioxidant activity of L-EGCG; enhanced availability of EGCG	2020	[176]
Ellagic acid (EA)	In vitro and in vivo	10 µg/mL	Liposomes (EA-Hb/TAT and isoDGR-Lipo)	170.77–212.90	Hyperglycemia/hypoxia-induced injury in ARPE-19 cells/HUVECs	Ameliorated retinal structure, antioxidative, downregulated the expression of GFAP, HIF-1α, VEGF, and p-VEGFR2	Better cellular uptake; potential as eye drops; co-loaded with Hb	2023	[180]
		5 mg/kg (i.v., once every 3 days for 6 weeks); as eye drops			db/db mice				
Lisosan G	In vivo	1 g kg/day (p.o.)	Lisosan G in liposomes (LipoLG)	~130	STZ-induced DR (mice)	Restored retinal function, downregulated typical molecular hallmarks of DR (oxidative stress, inflammation, glial reaction, apoptosis, VEGF expression, and BRB breakdown)	Good entrapment efficiency of Lisosan G, good storage stability	2023	[178]
Quercetin	In vivo	50, 200 mg/kg (p.o.)	Pegylated quercetin liposomes (Q-PEGL)	128.8 ± 18.05	STZ-induced diabetic nephropathy (rats)	FBG level ↓, antioxidative	Maintaining higher quercetin concentrations in plasma	2020	[175]
SNEDDS									
Resveratrol	In vitro	As eye drops	RSV-SNEDDS	<100	Rabbit corneal epithelial cell line (SIRC)	/	Improved solubility, stability, and bioavailability; reduced drug loss during storage	2024	[182]
Solid lipid nanoparticles									
Quercetin	In vivo	5 and 10 mg/kg (i.p. for 21 days)	Nano-formulation of quercetin (NQ)	157.1–528.2	STZ-induced DR (zebrafish)	Neuroprotective, ameliorated DR	Good bioavailability	2020	[183]
Polymeric nanocarriers									
Curcumin	In vivo	nCUR (20 mg CUR equivalent/kg/day, p.o.) with or without subcutaneous insulin (2 IU/rat/day)	PLGA-GA2-CUR nanoparticles (nCUR)	261	STZ-induced DR (rats)	Anti-inflammatory and anti-hyperglycemic; prevented diabetic cataracts and retinopathy	Well-tolerated, lower nanoparticle toxicity	2023	[188]
Lutein	In vitro	Micellar lutein (10 μM) or LNCs (10 μM lutein) (0, 3, 6, 12, 18, 24 h)	Double-layered chitosan–sodium alginate-based lutein nanocarrier (LNCs)	95 ± 5	H_2_O_2_/CoCl_2-_treated ARPE-19 cells	Anti-angiogenic, antioxidative	Increased cellular uptake, slowed and controlled lutein release; LNCs improved the cellular efficacy of lutein by curtailing oxidative stress.	2023	[186]
Lutein	In vitro and in vivo	0, 1, 5, 10, 15, 15, 20, or 50 μM for 24 h	Lutein-loaded chitosan–sodium alginate-based nanocarrier systems (LNCs)	98 ± 5	H_2_O_2_-treated ARPE-19 cells	Antioxidative	Longer half-life of lutein, higher bioavailability	2021	[185]
		600 μM (p.o.)			STZ-induced DR (rat)				
Resveratrol	In vitro	Intravitreal injection	PMs-Rv-Rh6G	3579 ± 0.19	HRPE cells (D407)	Anti-VEGF, anti-inflammatory	High-efficiency encapsulation of resveratrol	2019	[187]
Metal-based and biopolymeric nanoparticles									
Resveratrol	In vitro	/	NIR light-responsive thermoplasmonic-triggered release of therapeutic resveratrol-carrying polymeric microcapsules (MC)	<100	HRPE cells (D407)	Anti-VEGF	Light-triggered delivery and release; high stability; great biocompatibility	2022	[194]
Metal-based nanoparticles									
Ellagic acid (EA)	In silico docking study	/	Nano-encapsulated ellagic acid (NEA)	161–297	/	Inhibitory actions on aldose reductase and α-glucosidase	Improved the solubility and biological responses besides minimizing toxicity and degradation	2020	[190]
Rutin	In vivo	Rutin (10 mg/kg/day) or AuNPsR (0.6 mL/day); (p.o. for 7 days)	AuNPsR	8–22 (mean = 15)	STZ-induced DR (rats)	Antioxidative, improved fundus appearance of retinal arterioles, decreased MDA, and increased antioxidant capacity	Improved bioavailability, green synthesis	2023	[193]
Quercetin	In vitro and in vivo	Fe-Quer NZs (25, 50, 100, or 200 µg/mL) for 48 h	Ultrasmall Fe-Quer nanozymes (NZs)	5–10	HG-induced injury in HUVECs and monkey choroid–retinal endothelial cells (RF)	Protected against inflammation, oxidative stress damage, microvascular leakage, and angiogenesis	Exhibiting excellent water dispersibility and efficient ROS scavenging ability	2023	[191]
		60 mg/kg (p.o.)			STZ-induced DR (rats)				

Abbreviations: Administration routes: i.p., intraperitoneal injection; i.v., intravenous injection; p.o., per os (by mouth). Others: BRB, blood–retinal barrier; FBG, fasting blood glucose; GFAP, glial fibrillary acidic protein; Hb, hemoglobin; HIF-1α, hypoxia-inducible factor-1 alpha; p-VEGFR2, phosphorylated vascular endothelial growth factor receptor 2; STZ, streptozotocin; VEGF, vascular endothelial growth factor; ↓: down-regulated.

## 6. Conclusions and Further Remarks

Overall, ample evidence has demonstrated the promising potential of nutraceuticals in managing DR, either through oral administration, injections, or even through topical application. Meanwhile, scientists are also motivated to explore the underlying mechanisms of nutraceuticals in DR-related models. Indeed, most nutraceuticals display strong antioxidant and anti-inflammatory properties. Furthermore, some of them could protect neurons, neuroglia, and epithelial cells and improve retinal function and retinal vascular function. Such effects may involve different pathways like NF-κB and HIF-1α signaling, which can provide clues on the roles of nutraceuticals in DR.

However, when it comes to practical clinical utilization, some prominent candidates lost their magic, as indicated in some clinical trial studies, such as omega-3 PUFAs. Additionally, the relatively low bioavailability (low bioaccessibility, absorption rate, or transformation) of most nutraceutical-like carotenoids and polyphenols impedes their practical application. Moreover, some DM patients may have impaired liver and renal function, which prevents them from taking relatively large doses of nutraceuticals that are tested to be effective. However, the technique of nanoparticle delivery systems offers the benefits of greater carrying capacity and better bioavailability for nutraceuticals. Notably, the targeted and controlled-release modifications of nanoparticles have enabled tailored delivery of nutraceuticals to the retina. This is of great importance as many active nutraceuticals cannot reach high concentrations in the retina due to the blood–ocular barrier. However, the newly emerging products are mainly in their initial stages of research and development, with most studies being in vitro and few being in vivo; further research is crucial to provide important information for supporting and promoting their clinical application in NPDR and DR.

Advancements in nutraceutical delivery designs and industrialized production not only aid in disease prevention and patient recovery, but also meet market demands. According to Mordor Intelligence research company, the global nutraceutical market in 2024 is estimated to be USD 488.41 billion, with a compound annual growth rate of 5.09% from 2024 to 2029 [https://www.mordorintelligence.com/industry-reports/global-nutraceuticals-market-industry (accessed on 22 May 2024)]. The growing interest in functional foods among consumers stems mainly from increased health awareness and potential health benefits of nutraceuticals, as well as economic growth and customer spending. Thus, research on nutraceuticals is essential in view of their scientific and socio-economic impact.

In conclusion, despite their relatively low bioavailability and low efficiency compared to many drugs, nutraceuticals, especially when administrated orally, possess the advantages of being nontoxic and easily accessible with few complications. Nutraceuticals offer strong antioxidant and anti-inflammatory effects, making them potential retino-protective prophylactic daily supplementations for patients affected by NPDR and possibly PDR, improving their quality of life. With the power of nano-delivery technology, nutraceuticals can better benefit DR patients as a more effective, precise, and harmless adjunctive treatment strategy.

## Abbreviations

AGEs	Advanced glycation end products
AMPK	AMP-activated protein kinase
Ang-2	Angiopoietin 2
AuNPs	Gold nanoparticles
BRAs	Black raspberry-derived anthocyanins
BRB	Blood–retinal barrier
C3G	Cyanidin-3-glucoside
DHA	Docosahexaenoic acid
DME	Diabetic macular edema
DR	Diabetic retinopathy
EGCG	Epigallocatechin-3-gallate
eNOS	Endothelial nitric oxide synthase
EPA	Eicosapentaenoic acid
ER	Endoplasmic reticulum
ERK1/2	Extracellular signal-regulated kinase 1/2
FADH2	Flavin adenine dinucleotide
GIT	Gastrointestinal tract
GRP78	78-kDa glucose-regulated protein
HG	High glucose
HIF-1α	Hypoxia-inducible factor alpha
HO-1	Heme Oxygenase-1
HREC	Human retinal endothelial cells
IL	Interleukin
MAPK	Mitogen-activated protein kinases
mTOR	Mammalian target of rapamycin
MUFA	Monounsaturated fatty acids
NEs	Nanoemulsions
NF-κb	Nuclear factor-kappa B
NGF	Nerve growth factor
NLRP3	NOD-like receptor family pyrin domain containing 3
NPDR	Nonproliferative diabetic retinopathy
Nrf2	Nuclear factor erythroid 2-related factor 2
O/W	Oil-in-water
OA	Oleic acid
OIR	Oxygen-induced retinopathy
PDR	Proliferative diabetic retinopathy
PI3K	Phosphoinositide 3 kinase
PKC	Protein kinase C
PKR	Protein kinase R
PRP	Panretinal photocoagulation
PSD	Postsynaptic density
PUFA	Polyunsaturated fatty acids
RAGEs	Receptor for AGEs
RCT	Randomized controlled trial
ROS	Reactive oxygen species
RPE	Retinal pigment epithelial cells
SEDDS	Self-emulsifying drug delivery systems
SFA	Saturated fatty acids
SIRT1	Sirtuin 1
SIRT3	Sirtuin 3
SNEDDS	Self-nanoemulsifying drug delivery systems
STZ	Streptozotocin
T1DM	Type 1 diabetes mellitus
T2DM	Type 2 diabetes mellitus
TCA	Tricarboxylic acid
TNF-α	Tumor necrosis factor-α
TXNIP	Thioredoxin-interacting protein
VEGF	Vascular endothelial growth factor
VTDR	Vision-threatening diabetic retinopathy
W/O	Water-in-oil
